# Pork and Potatoes—Their Influence on the Bodies and Minds of Our People

**Published:** 1857-09

**Authors:** 


### *Pork and Potatoes: their influence on the Bodies and Minds of our People. Wisdom of the Mosaic Law.*

"Scrofula," from Scofa—a hog: see the Dictionaries.

The miserable and degrading mental feebleness with which the great mass of the people continue to ignore most subjects that effect their bodily health, until their ignorance overwhelms them with some great calamity, is certainly but ill calculated to give the health-teacher any high opinion of their intellect, or of the prospective value of his own labors to the public good. Our Lager Bier and Tobacco articles have raised such a storm amongst the elegant and intellectual votaries of those delicious luxuries, as to show pretty clearly the tenacity with which admirers will probably cling to the interesting quadruped of whose virtues we are now to discourse. Indeed, the amiable creature fairly reciprocates the compliment of their devotion to lager bier, by partaking of one portion of their favorite beverage, (it is true under the domestic inconvenience of somewhat contracted accommodations and a limited and compulsory bill of fare,) but with gratifying gusto and a still increasing fatness, so much appreciated by its biped admirer. There is an evident unkindness, however, yet we can not but think it creditable to the æsthetics of the quadruped, that he carries the compliment no further than the acceptance of a portion only of the lager bier, as a diet drink, and refuses tobacco in toto; a better physiologist than his master, he declines the intoxicating portion of the beverage, and can find none of his bodily wants supplied by smoke; the refuse of the still and the brewery, the poor fellow is constrained to take from necessity; but it passes the art of the great tyrant to make him chew or smoke; in that he surely shows the superiority of instinct over education; nor do we think we slander the gentlemen who improve their classic outlines, and dignify their steps by lager, when we compliment the quadruped for the superiority of his taste; it evidently serves him far better than that which cannot recognize the strong and acrid taste of the tobacco necessary to bring the precious beverage up to the gradually fuddling point, without which the consumer would loose his relish for it. We defy any man whose palate is sufficiently undefiled to relish a piece of wholesome bread or good beef, not to taste the acrid tobacco in many samples of the beverage; with a glass of Croton in hand ready to rinse the mouth, we have repeatedly detected it, and men of sound observation who have abandoned the pernicious drink, have often assured us that the effect of such drugged specimens is exactly analogous on the brain, to the first effect of tobacco before the system has become accustomed to it; put as much tobacco into his allowance of swill as would strengthen the lager bier up to the fuddling point, and you will soon observe the superiority of instinct over education; our life for it, the quadruped will give you an expressive glance of reproach, and refuse the proffered boon at once.

It seems, however, that this sagacity is not proof against the infernal machinations of his Western keepers; or what is more likely, that the tyranny of man has constrained the hog to eat what his instincts would teach him to refuse. We learn from our exchanges that great numbers of hogs have died in the neighborhood of Cincinnati, and indeed all over the West, from an epidemic disease analogous to cholera. Such has been the awful havoc amongst them, that we wrote to the editor of the *Erie City Dispatch* for reliable information, well knowing that we should get it from so fearless and independent quarter. Before we received his answer, which we give below, we learned that the Legislature of Ohio had passed a law rendering it a penal to put strychnine amongst the grain from which they distil whiskey! (it having been found that a vast increase of strength and intoxicating power may be given to liquor made by this process); the poor hogs were dying in vast multitudes from eating the swill, whilst the human consumer remained entirely unsuspicious of danger! Oh! oh! thought we, gentlemen of the Lager Bier trade in Cincinnati, amiable philosophers and good Christians, are you still preparing to drown our poor body in that barrel of lager, or to hoist it on that horrid pole? You have, it seems, a far more powerful enemy near home; the Legislative scalpel is too sharp for ye; ye will have to look out for some new dodge. Our friend of the *Dispatch* answered our inquiry by a letter detailing the losses at the various pens, to the number of tens of thousands, worth, when fat, six hundred thousand dollars!—He says: "We publish a short account of the Hog-Cholera to-day, from the Pittsburgh *Dispatch*, to which we ask the attention of our readers. We here wish to observe that but few people know, when they are eating pork, particularly that which has been fatted at large or in distilleries, the amount of disease and nastiness which they are taking into their stomach. You know that you are eating *hog*, but you do not know how much scrofula, erysipelas, and in many instances, the very elements of worse disease. Whole families sometimes live almost entirely on *hog*, and if the children are troubled with watery sores around the mouth, swelling and inflammation in the glands of the throat, the poor things have fever blisters, and have taken cold, which *settled in the neck!* or if they die with the croup, *Providence did it!* If anybody has ever killed a hog, and found an abscess of matter in the neck or near the kidneys, or with all the internal organs studded with tubercles, which we have seen packed for Eastern use, they have only put out of the world a miserable mass of scrofula; they did not kill anything fit to eat—nor would they, had the hog been healthy; and those who pride themselves on killing and eating fat hogs, must not be surprised if they swell up and burst. A fat hog is the quintessence of scrofula and carbonic acid gas; and he who eats it must not expect thereby to build up a sound physical organism. While it contributes heat, there is not a twentieth part of it nitrogen, the base of muscle."

This is sound practical truth. Fat pork was never designed for human food; it is material for breath and nothing more; see Liebig and other organic chemists and physiologists; it makes no red meat or muscle; the prize fighter is not allowed to eat it; all that is not consumed by the lungs, remains to clog the body with fat. As long ago as our 12th number we explained all this in an article entitled "Fat and its Uses." We cannot go over that ground again, but must be excused if we repeat in this place the sentiment advanced in that article, namely, that the man who is not acquainted with the elements of the body, and the actual value of the various articles of food that sustain it, occupies a position about as dignified as he who does not know the laws of his country, or who resigns the care of his soul to a Roman priest. The popular idea of boils and other unsightly eruptions being produced by an excess of grease in diet, is undoubtedly correct; notwithstanding the vital necessity of a good portion of wholesome beef and mutton, fat and butter, in all persons who are of low degree of life force or at all inclined to scrofula. We have most extensively treated that subject in many of our past numbers. Suffice it here to say, that fat is a vital necessity, and none can be healthy without it; but it requires a corresponding degree of exercise to throw it off by the lungs; its specific purpose in the economy is to supply material, that is, carbon, for breath, and to prevent the too rapid waste of the red meat or muscle of the body, which must and does take its place when the fat of the body is all consumed, and the individual eats none, or not enough to supply the waste by breathing.

We have said that fat never makes muscle, and we also aver that it does not give life force or capacity to endure fevers, and especially cholera. The carnivorous animal gets large supplies of nitrogen which is the basis of red meat that it eats; it has comparatively little fat, and it runs off that little very soon, and requires some fat in its food to produce more; this it gets from its prey; ready-made muscle is its food; the

Arab of the desert, and the American Indian are both lean from exercise; they rapidly consume their fat.

The hog, when fed by potatoes and kept quiet, makes little muscle but much fat; the Irish women are inclined to indolence, and eat excessively of patatoes; they are usually fat; it is the experience of every practical physician that they die rapidly from fevers and cholera, whilst the miserable lathy American stoutly resists both, and far oftener recovers; the puffiness and watery looking fatness of paupers and old prisoners who use a pure vegetable diet, and little or no exercise is remarkable; the cholera and typhus fever scourge the prisons awfully. We have seen eleven deaths in one morning, in the year '32, at Bellivue, of Cholera. Luxurious people are never strong. Activity and flesh eating give life force Indolence and vegetable eating decrease it. Whatever the cause may be of the tremendous mortality among the hogs, now existing at the West, it is very evident it is a deficiency of organic or contractile power, for they die of diarrhœa, and the general resemblance of the symptoms has given the disease the appelation of Hog Cholera.

The wisdom of the Jewish law which prohibits the use of pork, will soon be acknowledged by all rational beings. That swine are afflicted with scrofula and tubercles, we have repeatedly shown; and every killer of hogs well knows it; the indigestibility of the flesh is acknowledged, and if people were enlightened, the hog would only be raised for his fat alone; this is available in all cases instead of whale or fish oils, and will doubtless be made so in place of vegetable oils; a few excepted of the finer kinds for eating. Mutton and beef, if our farmers ever become enlightened, may profitably take the place of the hog, and would add greatly to the health and dignity of the farmers household. Hog husbandry is debasing. The influence of pork eating on the farmer is degrading; neither he nor the slave would be able to accomplish his work by the use of it, that is, the fat part, without other food, as material for muscle — Good husbandry would furnish other fats for the farmer, to say nothing of butter, which the present catalogue of the grasses and cereals, and enlightened irrigation, would furnish in sufficient abundance..

And this brings us to speak of the influence of pork eating on the moral condition of our people. If, and we emphatically aver it a correct criterion, the material and getting up of the family meal classifies the occupant of a household amongst rational and intellectual beings, then we say that those on whose tables pork is most frequently found, are the least intelligent and most groveling in their views. We know distinctly the full bearings of what we utter, and precisely how it will be received, nor do we care a farthing for the anathemas that will be hurled at us for the assertion. The potatoe has done more to demoralize and debase Ireland and keep the inhabitants under a vile and designing priesthood, than all the extravagance of monarchy and the harlotry of the English Church. Nor would the poor Irish wretches seek heaven in the whiskey-bottle and at the confessional, if their miserable bodies were not exhausted of organic force by an unnutricious root, which requires nothing but a pair of hands to claw it from the earth, a few bits of peat to cook it, and the hard earth of the cabin, foul and damp with human filth, and often shared as a lodging room with the foul animal we speak of, as a family board.—Dr. Pereira ranks it at only one-tenth the nourishment of meat! What motive remains for his elevation, if a human being is taught to look no higher than such a repast for the nourishment of his body, and to the nearest priest for the welfare of his soul? Now compare this fairly with the slave and his hog and ash-cake, and the poor farmer, such as we have often seen at the family meal—ay, as you may now find him all over our country, with his salt hog, potatoes, and sour rye bread, and his miserable husbandry, and thousand excuses for not raising sheep, protecting and feeding his cattle, and changing and cultivating his fowls and seeds, and attending carefully to a good vegetable garden. You need make but small search for his pig pen, his foul barn yard, his whiskey-bottle, and tobacco-box, or his nauseous pipe. He will largely defend his beloved porkers, his system (!) of husbandry and his favorite luxuries; the hog is his grand exemplar of manners and physiologies, his omnivorous animal laboratory that converts the produce of his farm into his great staple—pork. Poor Sir Walter Raleigh, we have often shed a tear for your sad fate; but our wicked fancy has often pictured ye making an offering to the devil of a hog stuffed with tobacco. The devils, we think, selected a very appropriate lodgment when they besought the Saviour to send them into the hogs; it is a great pity that the entire family of swine were not comprised by that same two thousand; we should have thanked Heaven for their devotion to hydropathy.—*Scalpel.*

